# Theoretical Mechanism on the Cellulose Regeneration from a Cellulose/EmimOAc Mixture in Anti-Solvents

**DOI:** 10.3390/ma15031158

**Published:** 2022-02-02

**Authors:** Zhaoyang Ju, Yihang Yu, Shaokeng Feng, Tingyu Lei, Minjia Zheng, Liyong Ding, Mengting Yu

**Affiliations:** 1 College of Chemical and Material Engineering, Quzhou University, Quzhou 324000, China; jzy@qzc.edu.cn (Z.J.); gongbenluoas@163.com (Y.Y.); fengshaokeng@126.com (S.F.); Zmj_1210@163.com (M.Z.); 2 Institute of Coal Chemistry, Chinese Academy of Sciences, Taiyuan 030001, China; tingyulei@hotmail.com

**Keywords:** cellulose, ionic liquids, regeneration, theoretical calculations

## Abstract

The experiments on cellulose dissolution/regeneration have made some achievements to some extent, but the mechanism of cellulose regeneration in ionic liquids (ILs) and anti-solvent mixtures remains elusive. In this work, the cellulose regeneration mechanism in different anti-solvents, and at different temperatures and concentrations, has been studied with molecular dynamics (MD) simulations. The IL considered is 1-ethyl-3-methylimidazolium acetate (EmimOAc). In addition, to investigate the microcosmic effects of ILs and anti-solvents, EmimOAc-*n*H_2_O (*n* = 0–6) clusters have been optimized by Density Functional Theory (DFT) calculations. It can be found that water is beneficial to the regeneration of cellulose due to its strong polarity. The interactions between ILs and cellulose will become strong with the increase in temperature. The H-bonds of cellulose chains would increase with the rising concentrations of anti-solvents. The interaction energies between cellulose and the anions of ILs are stronger than that of cations. Furthermore, the anti-solvents possess a strong affinity for ILs, cation–anion pairs are dissociated to form H-bonds with anti-solvents, and the H-bonds between cellulose and ILs are destroyed to promote cellulose regeneration.

## 1. Introduction

Plants and plant-based biomass are abundant sources of renewable feedstocks found across the Earth which contain three dominant components: cellulose, hemicellulose, and lignin [1]. Cellulose can be derived from cellulose-rich starting materials, such as trees, cotton, and crop wastes, with an annual yield of over 10^12^ tons [2]. Apart from its broad applications as a raw material to produce paints, tissues, paper, new structural–functional membranes, and pharmaceutical compounds, cellulose can be also used as an appropriate feedstock for biofuel and bioproducts [3,4]. Due to the global challenge of the environment and energy crisis, there is an intensive growth in demands for green and renewable energy sources. Thus, the utilization of renewable lignocellulose has shown great promise for the future.

Cellulose is a linear condensation polymer with degrees of polymerization (DP) from 100 to 20,000, consisting of repeated glucopyranose units linked by β-1,4-glucosidic bonds [5,6]. The hydroxyl groups along the chains are connected via hydrogen bonds (H-bonds), in both parallel and anti-parallel fashion, as a result of its structural robustness with strong mechanical strength. Therefore, cellulose usually cannot be dissolved in common solvents, such as alcohol or water [7]. Ionic liquids (ILs) are composed of ions with a melting point around or below 373 K [8]. There are many unique physicochemical properties for ILs, such as low vapor pressure, high chemical stability, a wide electrochemical window, a wide liquid range, and high solvation ability to dissolve various substances which are difficult to dissolve in conventional solvents, and this has led to numerous proposed applications in various fields, including catalysis, extraction, electrochemistry, organic synthesis, etc. [9,10]. In 2002, Rogers and co-workers reported the use of ILs as cellulose solvents for both physical cellulose dissolution and regeneration, opening up a new class of solvents to the cellulose research community [11]. Up to now, approximately 300 kinds of ILs have been tested experimentally for dissolving lignocellulosic biomass [12,13,14]. Generally, strong H-bonds can be formed between cellulose and anions, and the dissolution ability of cellulose depends on the strength of the H-bonds formed with anions [15]. Besides, cellulose can be regenerated from the dissolved cellulose–ILs mixtures when adding anti-solvents, such as water or alcohol [16,17,18]. Cellulose regeneration is one of the most important parts of the utilization of cellulose and its transformation into new functional materials.

The regeneration of cellulose by coagulation with anti-solvents is an important pathway for the industrialization of cellulose materials. The regenerated cellulose has exhibited good mechanical properties, and can be used in fiber manufacture, films/membranes, hydrogels/aerogels, etc. [19,20]. Yousefi et al. reported that an all-cellulose nanocomposite was directly fabricated using the nano-welding of cellulose microfibers as a starting material in a BmimCl solvent [21]. In addition, Gupta and co-workers studied cellulose regeneration from a cellulose/BmimOAc mixture through the addition of anti-solvents by using molecular dynamics (MD) simulations, and concluded that water is identified as one of the best potential candidates for cellulose regeneration [22]. Shamsuri et al. summarized the properties and applications of cellulose regenerated from cellulose/imidazolium-based ILs/co-solvent solutions, and provided a good understanding of the changes in the properties of regenerated cellulose blends [23]. Most of the studies are focused on the yield and properties of cellulose regeneration. However, the microcosmic mechanism of cellulose regeneration, the roles of anti-solvents, and the interactions between ILs and anti-solvents have not been revealed yet.

With the rapid development of computer science, computational modeling methods, including MD and Density Functional Theory (DFT) calculations, have been successfully used in the dissolution of lignocellulose and the conversion mechanism of biofuels [24,25]. Miyamoto et al. investigated the structural reorganization of two different molecular sheets derived from cellulose II crystal using MD simulations, and proposed that the van der Waals-associated molecular sheet will be stable because of its hydrophobic inside and hydrophilic periphery [26]. Some studies also recognized that insolubility is often attributed to the strong inter- and intramolecular H-bonds between cellulose molecules through a computational perspective [7,27]. The mechanism of regeneration from the cellulose–ILs mixtures to their aggregation state is essential for the successful preparation of the regenerated cellulose materials, which dominates their morphology and properties. The physical, chemical, and electrochemical properties will be changed when added to water, or organic solvents in pure ILs. Chaban et al. had summarized the fundamental research and prospective application of ILs in combination with molecular liquids, concentrating on the viscosity of ILs, micelle formation, and the adsorption of pollutant gases [28]. Additionally, they investigated the conductivity of imidazolium-based ILs and the acetonitrile binary system through MD simulation, and they found that acetonitrile can boost the conductivity and diffusion of the ILs/acetonitrile mixture [29]. As one kind of efficient IL for cellulose dissolution and regeneration, 1-ethyl-3-methylimidazolium acetate (EmimOAc) has been widely studied in the dissolution and regeneration of lignocellulose [30,31], but the corresponding mechanism of cellulose regeneration in EmimOAc/anti-solvent mixtures remain ambiguous.

In this work, the role of anti-solvents, such as water, methanol, and ethanol, in cellulose regeneration has been considered for cellulose regeneration from cellulose/EmimOAc mixtures through a large-scale MD simulation. Besides, we have also investigated the temperature and concentrations of anti-solvents, which can affect the cellulose regeneration. H-bonds, the interaction energies of cellulose–ILs, and radial distribution functions have been analyzed to reveal the mechanism of cellulose regeneration. To further study the microcosmic interactions between ILs and anti-solvents, DFT calculations have been made for the EmimOAc-*n*H_2_O clusters. A preliminary study about the regenerated mechanism of cellulose in an EmimOAc/anti-solvent mixture has been conducted and the microcosmic mechanism of the regeneration of cellulose was revealed, and it provides some theoretical foundations for the utilization of cellulose.

## 2. Computational Methods

### 2.1. Molecular Dynamics Simulations

**Figure 1:**
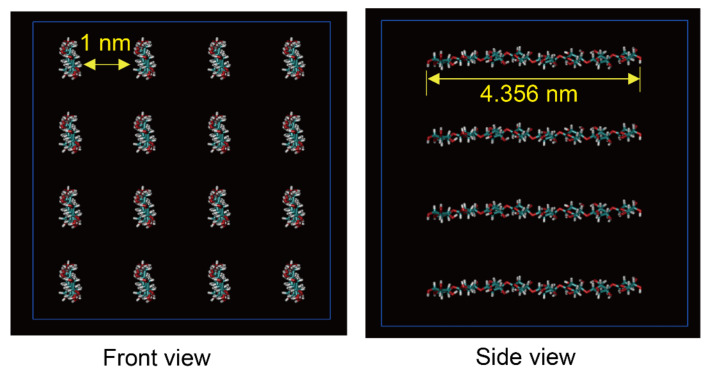
The initial configuration of cellulose (16 × 8) with 16 glucan chain system and a degree of polymerization (DP) = 8.

MD simulations for a cellulose/EmimOAc system, with 16 × 8 (16 glucan chains and each with 8 residues) cellulose bunches (Figure 1) and 320 pairs of EmimOAc filled in a cuboid box using Packmol [32], were performed with the Gromacs 5.1.1 software package. The force field parameters for EmimOAc are obtained from Liu’s work [33] with the spirit of AMBER framework. The Glycam06 force field [34] was used for cellulose bunches, and the SPC/E model [35] is used for water. The solvated cellulose was regenerated in a cuboid box filled with equilibrated ILs and solvents. Periodic boundary conditions (PBC) were used, with an initial box size of 7 nm × 7 nm × 7 nm in the x, y, z directions. All the covalent bonds were constrained using the LINCS algorithm [36]. The particle mesh Ewald summation was used in the calculation of long-range electrostatic interactions, with a cutoff radius of 1.2 nm. Firstly, the initial configurations were minimized by the steepest descent method until reaching the 100 kJ/mol·nm minimum force needed to remove the atomic collision. Then, the systems were equilibrated under an NVT ensemble for 500 ps, with a V-rescale thermostat at room temperature. Another 5 ns of annealing was used to increase the temperature from 298 to 400 K, consistent with the experimental dissolution condition [37]. In the above equilibrations, a harmonic restraint potential (a force constant of 1000 kJ/mol·nm^2^) was set on all carbon atoms to keep the cellulose’s initial position. Finally, a production run of 100 ns was performed with restraints removed in the NPT ensemble with a 1 fs timestep. Every 100 ps was used to collect the atomic coordinates, velocities, and energies.

### 2.2. Quantum Chemistry Calculations

The interactions of the EmimOAc-*n*H_2_O (*n* = 0–6) clusters were calculated with the Gaussian 16 package [38]. The structural optimizations were carried out at the M06-2X/def2SVP theoretical level, which was preferred to be a general DFT method for describing nonbonded interactions [39,40]. Vibrational frequencies were also calculated to verify the stationary structure for all the configurations. The binding energies for the clusters of EmimOAc-*n*H_2_O (*n* = 0–6) are defined as follows:

(1) $$\Delta E_{b}=E\left(clusters\right)-E\left(anion\right)-E\left(cation\right)-nE\left(H_{2}O\right)+\Delta E_{BSSE}$$

To further understand the bond properties of the EmimOAc-*n*H_2_O (*n* = 0–6) clusters, a natural bond orbital (NBO) analysis was performed by the NBO program. Depending on the results of the NBO analysis, the second-order perturbation stabilization energy E(2) with the delocalization of *i*→*j* is estimated as:

(2) $$E\left(2\right)=q_{i}\frac{F{\left(i,j\right)}^{2}}{\varepsilon_{i}-\epsilon_{j}}$$

## 3. Results and Discussion

### 3.1. The Cellulose Regeneration in Different Anti-Solvents

**Figure 2:**
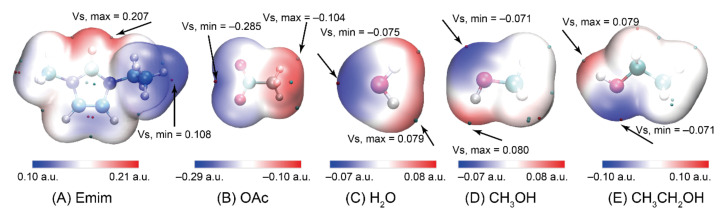
The electrostatic potential on the van der Waals surfaces of (**A**) Emim, (**B**) OAc, (**C**) H_2_O, (**D**) CH_3_OH, and (**E**) CH_3_CH_2_OH.

To characterize the structural properties of the anti-solvents, a quantitative molecular surface analysis of the electronic potential (ESP) has been widely used, based on the electrochemical structure [41,42]. In a molecular system, the ESP can be defined as:

(3) $$V_{tot}\left(r\right)=V_{nuc}\left(r\right)+V_{ele}\left(r\right)=\underset{A}{\sum }\frac{Z_{A}}{\left|r-R_{A}\right|}-\int \frac{\rho \left(r^{\prime }\right)}{\left|r-r^{\prime }\right|}dr^{\prime }$$

where *Z* and *R* are the nuclear charge and nuclear position, respectively. Additionally, *ρ*(*r*) is the electronic density function of the molecule. The ESP, mapped along with surface extrema of the anions and cations of EmimOAc and anti-solvents, are shown in Figure 2, and the graph of the surface area, plotted against different ESP ranges, is listed in Figure S1. The largest positive value of Emim can be found in the C2-H (0.207 a.u.) of the imidazole ring. The most negative value of OAc is around the oxygen (−0.285 a.u.). The electronegativity of the oxygen of the anions is strong, and easily attacks the electropositive region of cations. The highest electronegativity values of H_2_O, CH_3_OH, and CH_3_CH_2_OH are −0.075, −0.071, and −0.071 a.u., respectively. The polarity of H_2_O is stronger than that of alcohols. From Figure S1, there is a large portion of the molecular surface with a small ESP value in OAc, namely from −160 to −140 kcal/mol. H-bonds can be formed between cellulose and ILs that are beneficial for cellulose dissolution. However, H-bonds can be also formed between ILs and anti-solvents. Therefore, the H-bonds formed between cellulose and ILs will be weakened to promote cellulose regeneration.

**Figure 3:**
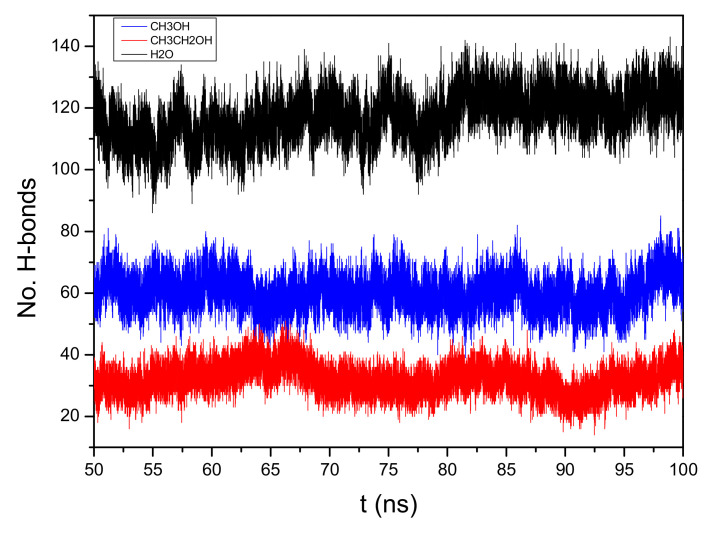
The number of H-bonds between cellulose chains in different 80 wt% anti-solvents in 293 K.

There are plenty of hydroxyls in the glucose unit of cellulose, and the inter- and intra-chain H-bonds could be formed as with the cellulose regeneration, which is shown in Figure S2. Two geometrical criteria were defined for the H-bonds: (1) the distance of the donor–acceptor is ≤3.5 Å, and (2) the angle of the hydrogen donor–acceptor is less than 30° (the deviation of the OH from the O--O internuclear axis) [43,44]. The H-bonds’ networks of cellulose are important standards against which to measure its regeneration [45]. To investigate the role of anti-solvents, the number of H-bonds between cellulose chains over 50–100 ns are collected in Figure 3. The number of H-bonds between the cellulose chains in the EmimOAc/water, EmimOAc/CH_3_OH, and EmimOAc/CH_3_CH_2_OH mixtures are around 120, 60, and 30, respectively. It means that the sequence of cellulose regeneration in the anti-solvent is H_2_O > CH_3_OH > CH_3_CH_2_OH.

### 3.2. The Effects of Temperature on Cellulose Regeneration

**Figure 4:**
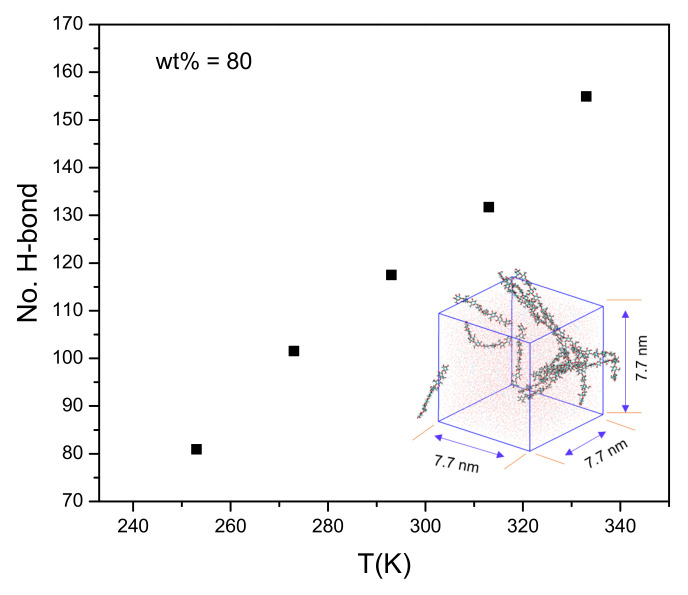
The (50–100 ns) average number of H-bonds between cellulose chains and ILs in different temperatures (80 wt% water).

There are many factors affecting the regeneration of cellulose, such as the anti-solvents’ concentration, temperature, and times [46,47,48]. To investigate the effects of temperature on cellulose regeneration, the (50–100 ns) average number of H-bonds between cellulose chains and ILs in 80 wt% water at different temperatures are shown in Figure 4. The distance between any two atoms of cellulose chains (<0.35 nm) was adopted as a geometrical criterion. The number of intra-chain H-bonds is one order of magnitude larger than that of the inter-chain, and the primary contribution to the cellulose bunches is from intra-chain interactions. The average numbers of H-bonds between the cellulose and ILs at 253 K, 273 K, and 333 K are 82, 103, and 155, respectively. The number of intra-chain H-bonds is one order of magnitude larger than that of the inter-chain, and the primary contribution to the cellulose bunches is from intra-chain interactions [22,49]. As shown in Figure S3, the H-bonds are relatively stable from 50 to 100 ns in the equilibrium system. The numbers of H-bonds at 253 K and 273 K are around 80 and 100, respectively. As the temperature is raised, the number of bonds to the ILs increases, which means the number of inter- and intramolecular H-bonds of cellulose decreases. It will not be beneficial for the regeneration of cellulose.

**Figure 5:**
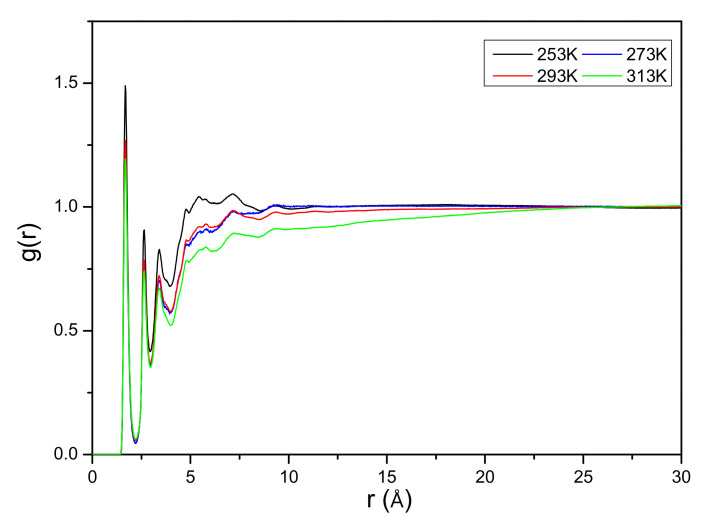
Radial distribution functions for cellulose around O atoms of OAc in different temperatures (80 wt% water).

To characterize the structural features of the effect of temperature on cellulose regeneration, radial distribution functions *g*(*r*) were calculated from the MD simulations using Equation (4):

(4) $$g_{ij}\left(r\right)=\frac{N_{ij}\left(r,r+△r\right)V}{4\pi r^{2}△rN_{i}N_{j}}$$

where *r* represents the distance between *i* and *j* atoms, *N_i_* and *N_j_* represent the numbers of *i* and *j* atoms, *N_ij_*(*r*, *r*+Δ*r*) represents the number of *j* atoms around *i* within a shell from *r* to *r*+Δ*r*, and *V* is the volume of the system. The graph in Figure 5 plots the *g*(*r*) for cellulose around the oxygen of OAc in the cellulose/EmimOAc/H_2_O mixtures. As shown in Figure 5, we find that OAc is mainly located around 1.5 Å to the hydroxyl group of cellulose, reflecting a strong interaction between cellulose and OAc. Besides, the interaction energies between cellulose and OAc are around −4360.37 kJ/mol at 253 K, −3933.06 kJ/mol at 273 K, −3711.84 kJ/mol at 293 K, and −3683.78 kJ/mol at 313 K, respectively. Upon increasing the temperature, the peak height drops, which implies the weakening of cellulose–OAc interaction strength based on the interaction analysis. It also confirmed that it will be not beneficial for cellulose regeneration with the increasing temperature.

### 3.3. The Effect of Water Concentration on Cellulose Regeneration

**Figure 6:**
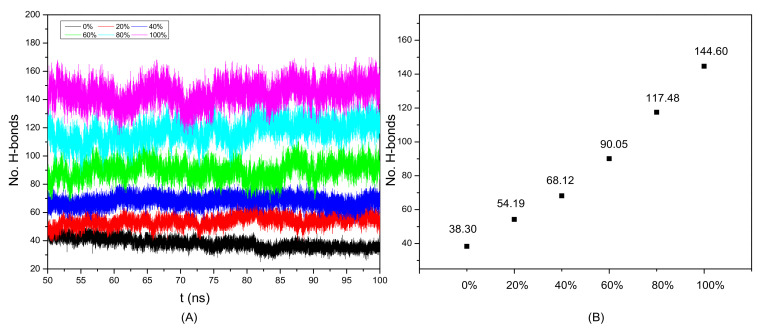
(**A**) The number of H-bonds and (**B**) average (50–100 ns) number of H-bonds between cellulose chains in the cellulose/EmimOAc/water mixtures at 20, 40, 60, 80, and 100 wt% water.

The different concentrations of anti-solvents play a critical role in the regeneration of cellulose [49]. To investigate the effects of water concentration on cellulose regeneration, we have summarized the number of cellulose H-bonds in 50–100 ns, which is shown in Figure 6. The weight percentage of solvents are defined as:

(5) $$\mathrm{wt}\%\;\mathrm{solvent}\;=\frac{\mathrm{weight}\;\mathrm{of}\;\mathrm{solvent}\;}{\mathrm{weight}\;\mathrm{of}\;\mathrm{solvent}+\mathrm{weight}\;\mathrm{of}\;\mathrm{ILs}}\times 100\%$$

As shown in Figure 6A, the H-bonds are relatively stable from 50 to 100 ns in the equilibrium system. The numbers of H-bonds in 60 and 80 wt% are around 90 and 120, respectively. In addition, we have calculated the average numbers of H-bonds in cellulose, which is shown in Figure 6B. The average numbers of H-bonds in 0, 20, 40, 60, 80, and 100 wt% are 38.3, 54.19, 68.12, 90.05, 117.48, and 144.60, respectively. Therefore, the numbers of H-bonds in cellulose will be increased with the rising of the water concentration. The cellulose will be reunited in the anti-solvents, and this will be beneficial for cellulose regeneration.

As the anions and cations of ILs play critical roles in the regeneration of cellulose, the interaction energy values help us to understand the regeneration process in the EmimOAc–water mixture. To investigate the interactions between cellulose and ILs, the interaction energies between the cellulose chains and the anion/cation of EmimOAc are listed in Table 1. Taking the interaction energies between the cellulose chains and ILs in 20 wt% water, for example, the interaction energy between the cellulose chains and Emim is −9764.80 kJ/mol and −17,511.8 kJ/mol for the OAc. The Lennard–Jones (LJ) of cellulose–Emim is −6302.39 kJ/mol, which is stronger than that of the coulombic interaction (−3462.37 kJ/mol). We found that the interaction energies of anions are stronger than that of the cations. Besides, the energy for the cations mainly comes from the LJ potential, while the energy for OAc mainly comes from coulombic interactions due to the small molecular volume of the anions. The contact area between the cellulose and cations is larger than that of the anions because of the bigger molecular dimension. Besides, the charge on the cations is more delocalized than that of the anions, based on the ESP analysis discussed above. Moreover, taking the interaction energies in 20 and 40 wt% water, for example, the interaction energy between the cellulose and anions are −17,511.8 kJ/mol and −14,104.60 kJ/mol, respectively. The interaction energy between the cellulose and cations are −9764.80 kJ/mol and −8514.32 kJ/mol, respectively. Therefore, as the water concentration increases, the number of interactions between the cellulose and both the anions and cations decreases. This contributes to the regeneration of cellulose with the increasing addition of water. Furthermore, the decrease in interactions between cellulose and the ILs is compensated by the increasing interactions between the ILs and water.

### 3.4. DFT Study on the Interaction between ILs and Anti-Solvents

**Figure 7:**
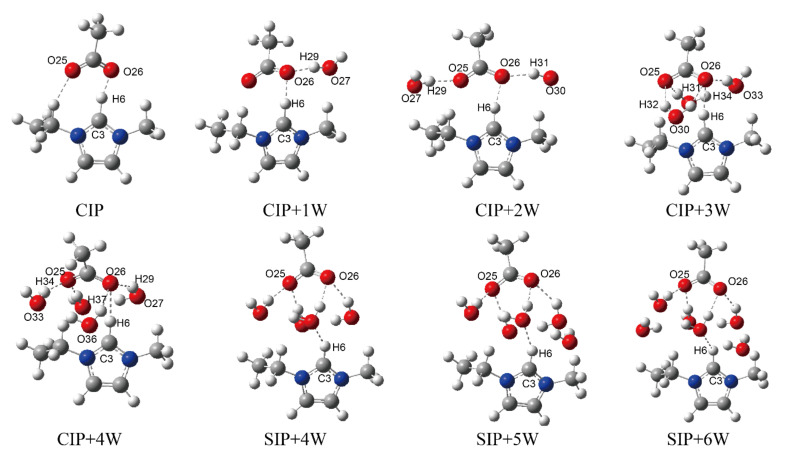
Density functional calculated structures of the solvent-separated ion pairs (SIPs) and contact ion pairs (CIPs) for EmimOAc-*n*H_2_O (*n* = 0–6) clusters.

**Figure 8:**
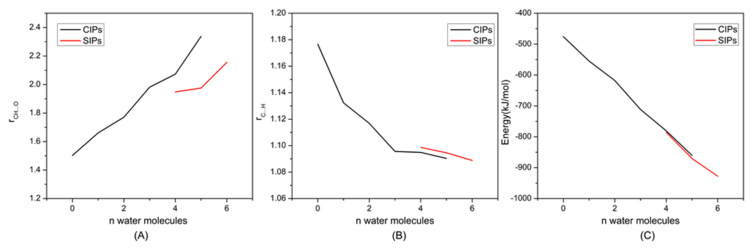
DFT calculated (**A**) intermolecular bond r_CH…O_(anion) and r_CH…O_(water); (**B**) intramolecular bond r_C…H_ of cation; (**C**) total binding energies for EmimOAc-*n*H_2_O (*n* = 0–6) clusters.

The H-bonds formed between the hydroxyls and both the anions and cations are the driving force for cellulose dissolution, and play a critical role in the regeneration of cellulose in ILs–anti-solvents [30,50,51]. To further investigate the interactions between ILs and anti-solvents, we had calculated the microstructures of EmimOAc-*n*H_2_O (*n* = 0–6) through DFT calculations. There are two kinds of dominant structures in ILs–anti-solvents, which are contact ion pairs (CIPs), with strong anion–cation interactions, and solvent-separated ion pairs (SIPs), with strong ion–solvent interactions [52,53]. The optimized structures of EmimOAc-*n*H_2_O (*n* = 0–6) are shown in Figure 7. Initially, CIPs are the dominant structures due to the strong electrostatic interaction between Emim and OAc. With the increasing numbers of water molecules, SIPs are the prevailing structures when *n* ≥ 5. Besides, the corresponding structural and energetic properties of these configurations are presented in Figure 8. For the CIP structure, strong H-bonds can be formed between Emim and OAc, with r_CH…O_ about 1.50 Å (Figure 8A). When *n* = 1, 2, and 3 in the EmimOAc-*n*H_2_O (*n* = 0–6) clusters, the r_CH…O_ are 1.66, 1.77, and 1.98 Å, respectively. Therefore, it can be found that the H-bonds between anions and cations will be decreased by adding the water molecules. For the intramolecular bond r_C…H_ of Emim in the EmimOAc-*n*H_2_O (*n* = 0–6) clusters (Figure 8B), the r_C…H_ of Emim when *n* = 1, 2, and 3 are 1.18, 1.13, and 1.12 Å, respectively. The C2-H bond length of the imidazole ring will be decreased with the rising number of H_2_O. Furthermore, the calculated binding energies for all of the configurations are shown in Figure 8C. The binding energies of EmimOAc-*n*H_2_O (*n* = 0–6) are corrected by the BSSE (basis set superposition error), which is listed in Table S1. The binding energy of EmimOAc is −440.8 kJ/mol. When *n* = 1, 2, and 3 in the EmimOAc-*n*H_2_O (*n* = 0–6) clusters, the binding energies are −528.7, −570.2, and −622.3 kJ/mol, respectively. From the analysis of the binding energies of the EmimOAc-*n*H_2_O (*n* = 0–6) clusters, the EmimOAc tended to be a more stable structure, with high hydration in an aqueous solution. For the configurations with four or more water molecules, the SIPs are more favorable in EmimOAc–anti-solvents. Besides, the SIP structures can provide better H-bond possibilities, and more OH proton donors of H_2_O can be involved in H-bonded structural motifs.

To study the bonding properties in the EmimOAc-*n*H_2_O clusters, the natural bond orbital (NBO) method [54] has been used to characterize H-bonds in terms of hyper-conjugative donor–acceptor interactions. Table 2 shows the main donor–acceptor interactions between Emim and OAc, as well as their second-order perturbation stabilization energies (E(2)). The strength of the donor–acceptor interaction can be denoted by E(2), and the larger E(2), the stronger interaction will be. From Table 2, the obvious and efficient overlaps can be found between the lone-pair orbitals of OAc and the anti-bonding orbital of Emim. The anion and cation can form a strong interaction, since the E(2) values of LP O26→σ* C3-H6 is 54.43 kcal/mol. With the increase in the number of water molecules, the interactions between anions and cations would decrease. Taking the CIP+1W as an example, the E(2) values of LP O26→σ* C3-H6 is 18.63 kcal/mol. The E(2) values show that the H-bonds between Emim and OAc become weak when adding water molecules. Taking the CIP+2W as an example, the E(2) value of LP O26→σ* O30-H31 is 18.47 kcal/mol. It is indicated that strong H-bonds can be formed between OAc and H_2_O. Therefore, it has been confirmed that it will be beneficial for cellulose regeneration due to the strong H-bonds formed between OAc and H_2_O when adding the water anti-solvent.

## 4. Conclusions

The regeneration of the cellulose bunches of 16 glucan chains, each with eight glucose residues (16 × 8), in EmimOAc/anti-solvents mixture was investigated using molecular dynamics (MD) simulations. We have considered the influence of different kinds of anti-solvents, temperatures, and concentrations on cellulose regeneration. To further investigate the microscopic interactions between EmimOAc and water solvents, a series of EmimOAc-*n*H_2_O (*n* = 0–6) clusters has been studied using Density Function Theory (DFT) calculations. Compared to the alcohol solvents, the effect of cellulose regeneration in water is better than that of other alcohols because of the strong polarity of H_2_O. Upon increasing the temperature from 253 to 333 K, the interaction energies between cellulose and ILs will increase with the rise in temperature, and it is not beneficial for cellulose regeneration. Furthermore, the number of H-bonds in cellulose chains will increase with the rising concentration of anti-solvents. The interaction energy of cellulose–OAc is stronger than that of cellulose–Emim, and the energy for OAc mainly comes from coulombic interactions due to the small molecular volume of the anion. EmimOAc tended to be a more stable structure with high hydration in an aqueous solution. Besides, the SIP structures can provide better H-bonds possibilities, and more OH proton donors of H_2_O can be involved in H-bonded structural motifs. It hindered the interactions between cellulose and ILs due to the H-bonds formed between the ILs and anti-solvents.

Overall, insightful structural and dynamic properties for cellulose regeneration in EmimOAc/anti-solvent mixtures at a microscopic level are provided in this work. These would provide some help to understand the mechanism of cellulose in ILs and anti-solvents.

## Figures and Tables

**Table 1:** Interaction energies (kJ/mol) between cellulose chains and anion/cation of EmimOAc in different water concentrations.

Entry	Emim			OAc		
	E_coul_	E_L–J_	E_total_	E_coul_	E_L–J_	E_total_
0%	−3796.20	−7644.57	−11,440.70	−23,310.90	−136.08	−23,446.98
20%	−3462.37	−6302.39	−9764.80	−17,095.40	−416.39	−17,511.8
40%	−3150.73	−5363.59	−8514.32	−13,431.40	−673.23	−14,104.60
60%	−1840.56	−3099.88	−4940.40	−7357.76	−669.81	−8027.57
80%	−899.75	−1378.85	−2278.60	−3532.38	−400.68	−3933.06

**Table 2:** The main electron donor–acceptor interactions in the EmimOAc-*n*H_2_O conformers and their second-order perturbation stabilization energies (E(2)) at M06-2X/def2SVP level.

Structure	Donor (i)	Acceptor (j)	E(2) kcal/mol	ε(i)-ε(j)	F(i,j)
CIP	LP O26	σ* C3-H6	54.43	0.85	0.194
CIP + 1W	LP O26	σ* C3-H6	18.63	0.80	0.111
	LP O26	σ* O27-H29	16.71	1.25	0.129
CIP + 2W	LP O26	σ* C3-H6	16.07	0.84	0.105
	LP O26	σ* O30-H31	18.47	1.28	0.138
	LP O25	σ* O27-H29	13.25	1.28	0.117
CIP + 3W	LP O26	σ* C3-H6	7.42	0.86	0.057
	LP O26	σ* O33-H34	16.42	0.79	0.110
	LP O26	σ* O30-H31	1.95	0.87	0.038
	LP O25	σ* O30-H32	5.86	0.88	0.066
CIP + 4W	LP O26	σ* C3-H6	6.24	0.88	0.067
	LP O26	σ* O27-H29	4.81	1.27	0.070
	LP O26	σ* O36-H37	1.29	1.27	0.036
	LP O25	σ* O33-H34	10.85	1.21	0.103
	LP O25	σ* O30-H32	30.99	0.89	0.150

## Data Availability

Data is contained within the article or Supplementary Materials.

## Supplementary Materials

The following supporting information can be downloaded at: https://www.mdpi.com/article/10.3390/ma15031158/s1, Figure S1: ESP mapped molecular vdW surface and area percent of Emim, OAc, H2O, CH3OH and CH3CH2OH; Figure S2: Intrachain (black dotted) and interchain (red dotted) H-bonds and H-bond criteria; Figure S3: The number of H-bonds between cellulose chains from 50 to 100 in different temperature. (80 wt% anti-solvents); Table S1: The binding energies (ΔEb) corrected by BSSE(ΔEBSSE) of EmimOAc-*n*H2O (*n* = 0–6).

## References

[1] Zhang Z., Song J., Han B. (2017). Catalytic Transformation of Lignocellulose into Chemicals and Fuel Products in Ionic Liquids. Chem. Rev..

[2] Klemm D., Heublein B., Fink H.P., Bohn A. (2005). Cellulose: Fascinating Biopolymer and Sustainable Raw Material. Angew. Chem. Int. Ed..

[3] Focher B., Palma M.T., Canetti M., Torri G., Cosentino C., Gastaldi G. (2001). Structural differences between non-wood plant celluloses: Evidence from solid state NMR, vibrational spectroscopy and X-ray diffractometry. Ind. Crops Prod..

[4] Muhammad N., Man Z., Bustam M. (2011). Ionic liquid—A future solvent for the enhanced uses of wood biomass. Eur. J. Wood Wood Prod..

[5] O’Sullivan A.C. (1997). Cellulose: The structure slowly unravels. Cellulose.

[6] Zhu C., Xin H., Hong J., Wang H., Wang C., Ma L., Liu Q. (2021). Hydrocarbon Distribution of Cellulose Hydrogenolysis over Ru–MoOx/C Combined with HZSM-5. ACS Sustain. Chem. Eng..

[7] Gupta K.M., Jiang J. (2015). Cellulose dissolution and regeneration in ionic liquids: A computational perspective. Chem. Eng. Sci..

[8] Rogers R.D., Seddon K.R. (2003). Ionic Liquids—Solvents of the Future?. Science.

[9] Freudenmann D., Wolf S., Wolff M., Feldmann C. (2011). Ionic Liquids: New Perspectives for Inorganic Synthesis?. Angew. Chem. Int. Ed..

[10] Zakrzewska M.E., Bogel-Łukasik E., Bogel-Łukasik R. (2010). Solubility of Carbohydrates in Ionic Liquids. Energy Fuels.

[11] Swatloski R.P., Spear S.K., Holbrey J.D., Rogers R.D. (2002). Dissolution of Cellose with Ionic Liquids. J. Am. Chem. Soc..

[12] Wang H., Gurau G., Rogers R.D. (2012). Ionic liquid processing of cellulose. Chem. Soc. Rev..

[13] Zhang J., Wu J., Yu J., Zhang X., He J., Zhang J. (2017). Application of ionic liquids for dissolving cellulose and fabricating cellulose-based materials: State of the art and future trends. Mater. Chem. Front..

[14] Badgujar K.C., Bhanage B.M. (2015). Factors governing dissolution process of lignocellulosic biomass in ionic liquid: Current status, overview and challenges. Bioresour. Technol..

[15] Guo J., Zhang D., Duan C., Liu C. (2010). Probing anion–cellulose interactions in imidazolium-based room temperature ionic liquids: A density functional study. Carbohydr. Res..

[16] Wu J., Zhang J., Zhang H., He J., Ren Q., Guo M. (2004). Homogeneous Acetylation of Cellulose in a New Ionic Liquid. Biomacromolecules.

[17] Zhang H., Wu J., Zhang J., He J. (2005). 1-Allyl-3-methylimidazolium Chloride Room Temperature Ionic Liquid:  A New and Powerful Nonderivatizing Solvent for Cellulose. Macromolecules.

[18] Hauru L.K.J., Hummel M., King A.W.T., Kilpeläinen I., Sixta H. (2012). Role of Solvent Parameters in the Regeneration of Cellulose from Ionic Liquid Solutions. Biomacromolecules.

[19] Sayyed A.J., Deshmukh N.A., Pinjari D.V. (2019). A critical review of manufacturing processes used in regenerated cellulosic fibres: Viscose, cellulose acetate, cuprammonium, LiCl/DMAc, ionic liquids, and NMMO based lyocell. Cellulose.

[20] Wang S., Lu A., Zhang L. (2016). Recent advances in regenerated cellulose materials. Prog. Polym. Sci..

[21] Yousefi H., Nishino T., Faezipour M., Ebrahimi G., Shakeri A. (2011). Direct Fabrication of all-Cellulose Nanocomposite from Cellulose Microfibers Using Ionic Liquid-Based Nanowelding. Biomacromolecules.

[22] Gupta K.M., Hu Z., Jiang J. (2013). Cellulose regeneration from a cellulose/ionic liquid mixture: The role of anti-solvents. RSC Adv..

[23] Shamsuri A.A., Abdan K., Jamil S.N.A.M. (2021). Properties and applications of cellulose regenerated from cellulose/imidazolium-based ionic liquid/co-solvent solutions: A short review. e-Polymers.

[24] Ju Z., Xiao W., Yao X., Tan X., Simmons B.A., Sale K.L., Sun N. (2020). Theoretical study on the microscopic mechanism of lignin solubilization in Keggin-type polyoxometalate ionic liquids. Phys. Chem. Chem. Phys..

[25] Ju Z., Zhang Y., Zhao T., Xiao W., Yao X. (2019). Mechanism of Glucose–Fructose Isomerization over Aluminum-Based Catalysts in Methanol Media. ACS Sustain. Chem. Eng..

[26] Miyamoto H., Umemura M., Aoyagi T., Yamane C., Ueda K., Takahashi K. (2009). Structural reorganization of molecular sheets derived from cellulose II by molecular dynamics simulations. Carbohydr. Res..

[27] Medronho B., Lindman B. (2015). Brief overview on cellulose dissolution/regeneration interactions and mechanisms. Adv. Colloid Interface Sci..

[28] Chaban V.V., Prezhdo O.V. (2013). Ionic and Molecular Liquids: Working Together for Robust Engineering. J. Phys. Chem. Lett..

[29] Chaban V.V., Voroshylova I.V., Kalugin O.N., Prezhdo O.V. (2012). Acetonitrile Boosts Conductivity of Imidazolium Ionic Liquids. J. Phys. Chem. B.

[30] Li Y., Liu X., Zhang S., Yao Y., Yao X., Xu J., Lu X. (2015). Dissolving process of a cellulose bunch in ionic liquids: A molecular dynamics study. Phys. Chem. Chem. Phys..

[31] Zhang J., Zhang H., Wu J., Zhang J., He J., Xiang J. (2010). NMR spectroscopic studies of cellobiose solvation in EmimAc aimed to understand the dissolution mechanism of cellulose in ionic liquids. Phys. Chem. Chem. Phys..

[32] Martínez J.M., Martínez L. (2003). Packing optimization for automated generation of complex system’s initial configurations for molecular dynamics and docking. J. Comput. Chem..

[33] Liu Z., Huang S., Wang W. (2004). A Refined Force Field for Molecular Simulation of Imidazolium-Based Ionic Liquids. J. Phys. Chem. B.

[34] Kirschner K.N., Yongye A.B., Tschampel S.M., González-Outeiriño J., Daniels C.R., Foley B.L., Woods R.J. (2008). GLYCAM06: A generalizable biomolecular force field. Carbohydrates. J. Comput. Chem..

[35] Berendsen H.J.C., Grigera J.R., Straatsma T.P. (1987). The missing term in effective pair potentials. J. Phys. Chem..

[36] Hess B., Bekker H., Berendsen H.J., Fraaije J.G. (1997). LINCS: A linear constraint solver for molecular simulations. J. Comput. Chem..

[37] Diez V., DeWeese A., Kalb R.S., Blauch D.N., Socha A.M. (2019). Cellulose Dissolution and Biomass Pretreatment Using Quaternary Ammonium Ionic Liquids Prepared from H-, G-, and S-Type Lignin-Derived Benzaldehydes and Dimethyl Carbonate. Ind. Eng. Chem. Res..

[38] Frisch M.J., Trucks G.W., Schlegel H.B., Scuseria G.E., Robb M.A., Cheeseman J.R., Scalmani G., Barone V., Petersson G.A., Nakatsuji H. (2016). Gaussian 16.

[39] Mao Q., Ren Y., Luo K.H., van Duin A.C.T. (2017). Dynamics and kinetics of reversible homo-molecular dimerization of polycyclic aromatic hydrocarbons. J. Chem. Phys..

[40] Simeon T.M., Ratner M.A., Schatz G.C. (2013). Nature of Noncovalent Interactions in Catenane Supramolecular Complexes: Calibrating the MM3 Force Field with ab Initio, DFT, and SAPT Methods. J. Phys. Chem. A.

[41] Fu R., Lu T., Chen F.-W. (2014). Comparing Methods for Predicting the Reactive Site of Electrophilic Substitution. Acta Phys. Chim. Sin..

[42] Wang Z., Liu Y., Zheng B., Zhou F., Jiao Y., Liu Y., Ding X., Lu T. (2018). A theoretical investigation on Cu/Ag/Au bonding in XH2P⋯MY(X = H, CH3, F, CN, NO2; M = Cu, Ag, Au; Y = F, Cl, Br, I) complexes. J. Chem. Phys..

[43] Luzar A., Chandler D. (1996). Hydrogen-bond kinetics in liquid water. Nature.

[44] Dong K., Liu X., Dong H., Zhang X., Zhang S. (2017). Multiscale Studies on Ionic Liquids. Chem. Rev..

[45] Fan Z., Chen J., Guo W., Ma F., Sun S., Zhou Q. (2018). Anti-solvents tuning cellulose nanoparticles through two competitive regeneration routes. Cellulose.

[46] Shibata M., Teramoto N., Nakamura T., Saitoh Y. (2013). All-cellulose and all-wood composites by partial dissolution of cotton fabric and wood in ionic liquid. Carbohydr. Polym..

[47] Cai J., Wang L., Zhang L. (2007). Influence of coagulation temperature on pore size and properties of cellulose membranes prepared from NaOH–urea aqueous solution. Cellulose.

[48] Liu S., Zhang L., Sun Y., Lin Y., Zhang X., Nishiyama Y. (2009). Supramolecular Structure and Properties of High Strength Regenerated Cellulose Films. Macromol. Biosci..

[49] Gupta K.M., Hu Z., Jiang J. (2013). Molecular insight into cellulose regeneration from a cellulose/ionic liquid mixture: Effects of water concentration and temperature. RSC Adv..

[50] Parthasarathi R., Balamurugan K., Shi J., Subramanian V., Simmons B.A., Singh S. (2015). Theoretical Insights into the Role of Water in the Dissolution of Cellulose Using IL/Water Mixed Solvent Systems. J. Phys. Chem. B.

[51] Li Y., Liu X., Zhang Y., Jiang K., Wang J., Zhang S. (2017). Why Only Ionic Liquids with Unsaturated Heterocyclic Cations Can Dissolve Cellulose: A Simulation Study. ACS Sustain. Chem. Eng..

[52] Stange P., Fumino K., Ludwig R. (2013). Ion Speciation of Protic Ionic Liquids in Water: Transition from Contact to Solvent-Separated Ion Pairs. Angew. Chem. Int. Ed..

[53] Marcus Y., Hefter G. (2006). Ion Pairing. Chem. Rev..

[54] Glendening E.D., Landis C.R., Weinhold F. (2013). NBO 6.0: Natural bond orbital analysis program. J. Comput. Chem..

