# Supplementary material for: Theoretical Mechanism on the Cellulose Regeneration from a Cellulose/EmimOAc Mixture in Anti-Solvents
Source: Materials (Basel). 2022 Feb 2;15(3):1158. doi: 10.3390/ma15031158 (PMC8837949; doi:10.3390/ma15031158)
Supplement: Supplementary file 1 [file materials-15-01158-s001.zip › materials-1550461-supplementary.pdf]

Supplementary

Theoretical Mechanism on the Cellulose Regeneration from a Cellulose/EmimOAc Mixture in Anti-solvents

Zhaoyang Ju [1], Yihang Yu [1], Shaokeng Feng [1], Tingyu Lei [2], Minjia Zheng [1], Liyong Ding [1,*] and Mengting Yu [1,*]

[1] College of Chemical and Material Engineering, Quzhou University, Quzhou 324000, China; jzy@qzc.edu.cn (Z.J.); gongbenluoas@163.com (Y.Y.); fengshaokeng@126.com (S.F.); Zmj_1210@163.com (M.Z.)

[2] Institute of Coal Chemistry, Chinese Academy of Sciences, Taiyuan 030001, China; tingyulei@hotmail.com

* Correspondence: liyongding1988@163.com (L.D.); yumengting15@mails.ucas.edu.cn (M.Y.)

Contents

Figure S1: ESP mapped molecular vdW surface and area percent of ILs and anti-solvents.
Figure S2: Intrachain (black dotted) and interchain (red dotted) H-bonds and H-bond criteria.
Figure S3 The number of H-bonds between cellulose chains from 50 to 100 in different temperature. (80 wt% anti-solvents)
Table S1 The binding energies (ΔE_b) corrected by BSSE(ΔE_{BSSE}) of EmimOAc-$n$$H_2O$ (n=0-6).
Optimized geometries of DFT calculated EmimOAc-$n$$H_2O$ (n=0-6) clusters.

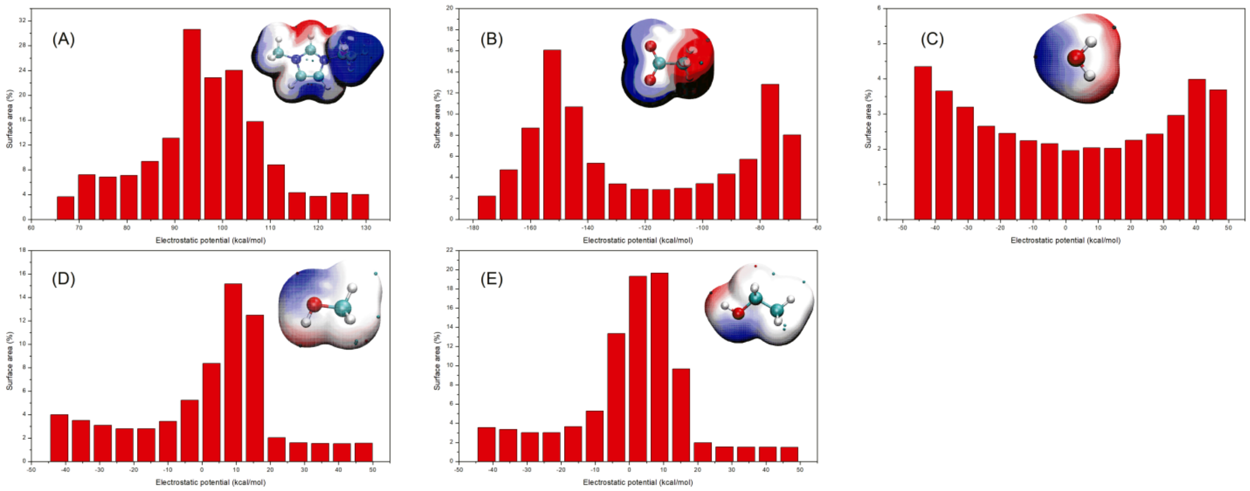

Figure S1. ESP mapped molecular vdW surface and area percent of (A) Emim (B) OAc (C) H_2O (D) CH_3OH (E) CH_3CH_2OH.

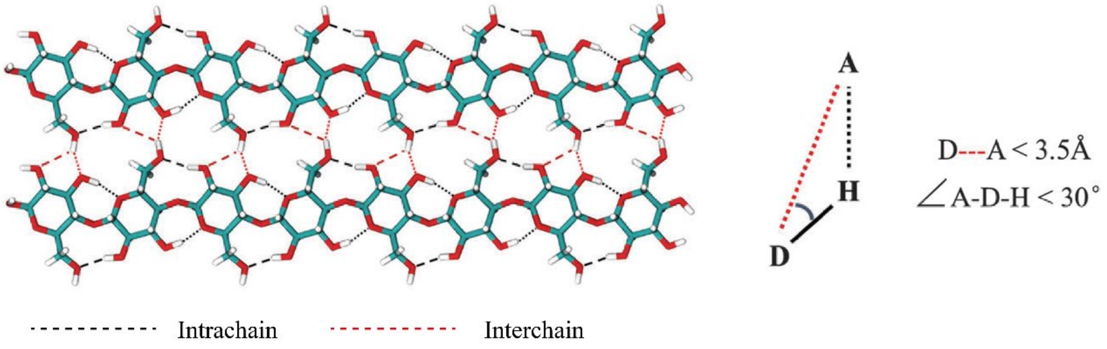

Figure S2. Intrachain (black dotted) and interchain (red dotted) H-bonds and H-bond criteria.

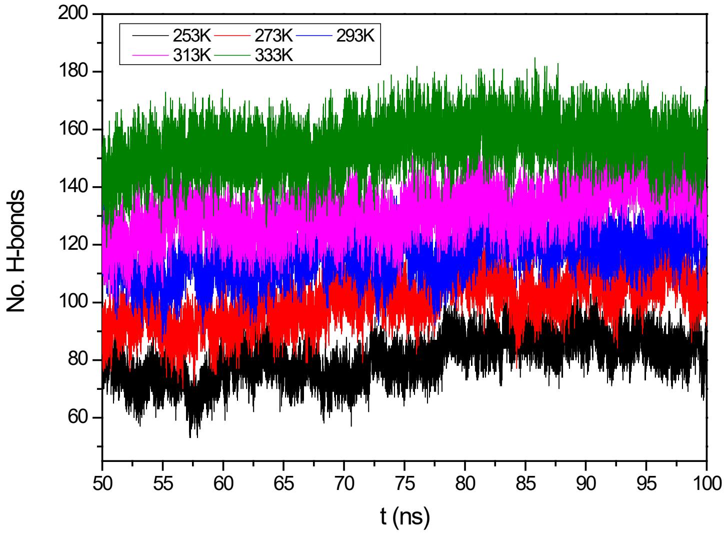

Figure S3. The number of H-bonds between cellulose chains from 50 to 100 in different temperature. (80 wt% anti-solvents).

Table S1. The binding energies (ΔE_b) corrected by BSSE(ΔE_{BSSE}) of EmimOAc-nH2O (n=0-6).

Structures	ΔE (kJ/mol)	ΔE_{BSSE} (a.u.)	ΔE_b (kJ/mol)
CIP	-475.7	0.0133	-440.8
CIP+1W	-554.9	0.0100	-528.7
CIP+2W	-617.7	0.0181	-570.2
CIP+3W	-711.6	0.0340	-622.3
CIP+4W	-780.5	0.0375	-682.1
SIP+4W	-795.5	0.0401	-690.3
CIP+5W	-860.3	0.0463	-738.7
SIP+5W	-870.4	0.0408	-763.4
SIP+6W	-927.9	0.0422	-817.2

Optimized geometries of DFT calculated EmimOAc-nH2O (n=0-6) clusters

CIP

0 1

C	2.63010300	0.19306500	-0.56637700
C	2.63491700	-1.15559800	-0.36719000
C	0.60556100	-0.39072900	0.12387600
H	3.41255300	0.86337400	-0.90882500
H	3.41986500	-1.89296000	-0.50202100
H	-0.53238500	-0.45283600	0.41645100
N	1.36139000	0.63897700	-0.26186800
N	1.36669300	-1.48843100	0.05928000
C	0.87742000	2.02425800	-0.32090400
H	1.33301000	2.48509300	-1.20832800
H	-0.21174900	1.94531900	-0.46969600
C	1.21940300	2.79033500	0.94628900
H	0.84791400	3.82034200	0.86477800
H	0.73518900	2.32356800	1.81510900
H	2.30442200	2.82701100	1.12413100
C	0.84721400	-2.81208500	0.37689600
H	1.30944800	-3.19311500	1.29631400
H	-0.23735500	-2.70648000	0.51572900
H	1.05136600	-3.49908200	-0.45336200
C	-4.07864300	-0.19149700	-0.23997200
H	-4.49648100	0.54197600	-0.93867600
H	-4.26951000	-1.21497800	-0.58984000
H	-4.56715500	-0.08772400	0.73996000

C	-2.57757400	0.02329800	-0.06464100
O	-2.05519600	1.04194000	-0.53840300
O	-1.96306800	-0.87939100	0.58696900

CIP+2W

0 1

C	2.17831500	2.21023100	-0.10387100
C	3.10240600	1.21178500	-0.01940500
C	1.10121500	0.28585500	-0.19416000
H	2.29272600	3.28969700	-0.09648500
H	4.18331200	1.24421800	0.07165600
H	0.30832700	-0.49948300	-0.23890700
N	0.94534300	1.61065200	-0.21824300
N	2.40652600	0.02570100	-0.07645200
C	-0.34327900	2.32288800	-0.27076700
H	-0.16508100	3.24338300	-0.84333100
H	-1.05326300	1.69222300	-0.81237100
C	-0.89015500	2.60545900	1.11536500
H	-1.86103600	3.10335700	1.00021200
H	-1.06736800	1.65121800	1.63031400
H	-0.21068700	3.23524000	1.70875100
C	2.98837400	-1.31318200	-0.03574700
H	3.64425600	-1.39015000	0.84001900
H	2.19198800	-2.06949000	0.03257500
H	3.57175200	-1.48813400	-0.94828200
C	-3.01081900	-2.35480700	0.15936200
H	-3.91082600	-1.80221200	0.45110200
H	-3.17868800	-2.85115200	-0.80731400
H	-2.79210100	-3.14206400	0.89308100
C	-1.81046200	-1.42619600	0.03002700
O	-1.98497500	-0.19106100	0.08217100
O	-0.68467200	-1.96299000	-0.14283600
O	-3.41842400	2.02590100	-0.49520400
H	-3.78767400	1.80065800	-1.35480200
H	-3.06339700	1.17011000	-0.17639100

O	1.11433400	-3.92094300	0.03231800
H	0.33587400	-3.34905000	-0.14294800
H	0.99513400	-4.15230600	0.95956200

CIP+3W

0 1

C	-3.03880700	0.30068800	-0.01010300
C	-3.07996100	-1.06040300	0.00861200
C	-0.97804800	-0.43044200	0.24398400
H	-3.83177200	1.03280400	-0.12142300
H	-3.91113000	-1.75195000	-0.08049300
H	0.10455900	-0.46157100	0.40942800
N	-1.72092500	0.66475900	0.14041300
N	-1.78029800	-1.49313400	0.16507300
C	-1.19468400	2.03388400	0.15154300
H	-1.58793700	2.52516000	1.05276200
H	-0.10851100	1.93910600	0.26771500
C	-1.54788400	2.77087800	-1.12847000
H	-1.11171200	3.77807600	-1.09659600
H	-1.12572800	2.22624000	-1.98340700
H	-2.63488500	2.88078200	-1.25782100
C	-1.33696500	-2.87887900	0.25701100
H	-2.02679300	-3.50354300	-0.32154600
H	-0.33237200	-2.94470900	-0.17941400
H	-1.33231800	-3.20520000	1.30475200
C	3.89172600	0.09950500	-0.32406300
H	4.39962100	1.06052900	-0.45802400
H	4.52768000	-0.61178800	0.21767700
H	3.67280500	-0.32992700	-1.31511600
C	2.56203100	0.27968100	0.39453500
O	2.05482000	1.41538400	0.43442300
O	2.01244700	-0.77560300	0.84153000
O	0.58679800	0.57119800	-1.97644800
H	1.17385600	1.06777300	-1.38651000

H	0.88553500	-0.35139400	-1.89872200
O	0.31410000	0.75996200	2.56698700
H	0.80438800	-0.06609400	2.43966600
H	0.86696000	1.33704300	2.01310500
O	1.35959200	-2.10776100	-1.26794900
H	1.74945300	-1.76545200	-0.41480800
H	2.04034400	-2.65217100	-1.67624600

CIP+4W

0 1

C	2.69396500	1.52774600	-0.37964300
C	3.42163700	0.40351800	-0.63065700
C	1.33587600	-0.19954100	-0.24948600
H	2.98782100	2.57191100	-0.35126100
H	4.47368400	0.26972700	-0.85963100
H	0.43265800	-0.80544000	-0.12349400
N	1.40130800	1.12322600	-0.14576100
N	2.55278200	-0.66164200	-0.54050300
C	0.25616200	1.98883000	0.15025300
H	0.06716800	2.60559500	-0.73792100
H	-0.60109400	1.32009400	0.26239900
C	0.47033700	2.80423400	1.40977900
H	-0.43313000	3.39967900	1.59713700
H	0.62884800	2.12233800	2.25482000
H	1.32142200	3.49610800	1.32580200
C	2.88739100	-2.06843400	-0.74185800
H	3.91607100	-2.23172100	-0.40103200
H	2.19005200	-2.66134200	-0.13265000
H	2.80390200	-2.32343000	-1.80583100
C	-3.70587400	-1.17530300	-0.68115100
H	-3.80396400	-0.32377900	-1.37247700
H	-3.75445800	-2.10887700	-1.25145800
H	-4.52331400	-1.11383500	0.04815600
C	-2.36249200	-1.03266900	0.01546300

O	-2.28560900	-0.14690900	0.91961800
O	-1.40649800	-1.72606300	-0.38213100
O	0.61146300	-2.82416300	1.20967200
H	0.49123400	-2.03788000	1.77270200
H	-0.17656500	-2.76210000	0.64325100
O	-0.05291100	-0.30364900	2.29755400
H	-0.30328500	-0.25727500	3.22627300
H	-0.92183600	-0.23888100	1.80162300
O	-2.71896300	2.01138400	-0.61367700
H	-2.73963100	1.33699500	0.10244600
H	-3.63588200	2.26015600	-0.76460100
O	-0.83462200	0.50414500	-2.19060500
H	-1.03419800	-0.39815500	-1.90367600
H	-1.54903300	1.04076400	-1.80637200

SIP+4W

0 1

C	3.57077200	0.73773000	0.16750800
C	3.87286300	-0.58726700	0.26374900
C	1.77795200	-0.41174400	-0.40892200
H	4.17050200	1.61972300	0.36836200
H	4.78752200	-1.09165900	0.55809800
H	0.75189900	-0.62875800	-0.73640400
N	2.26465100	0.81834000	-0.25871500
N	2.73981600	-1.28327500	-0.10031000
C	1.48816900	2.04820500	-0.46686700
H	1.99084200	2.62309600	-1.25805200
H	0.50677000	1.73433700	-0.83483300
C	1.32744400	2.85138800	0.80955900
H	0.66768500	3.70321500	0.60080600
H	0.84250600	2.21952300	1.56304100
H	2.28693400	3.22954700	1.19293900
C	2.60804200	-2.73654100	-0.16810200
H	3.12515000	-3.17538700	0.69309300
H	1.53575800	-2.97460100	-0.12320900

H	3.05691500	-3.10735100	-1.09837000
C	-4.90101700	-0.51167300	0.34148600
H	-5.32832200	0.40454500	-0.08957400
H	-5.34892100	-1.39463300	-0.12600600
H	-5.12394400	-0.49454700	1.41721400
C	-3.39317300	-0.50109700	0.14744100
O	-2.77525700	0.49302900	0.64397900
O	-2.85973500	-1.43784300	-0.47032600
O	-0.37275500	-2.44201300	0.33574700
H	-0.31190700	-1.63886900	0.88506000
H	-1.27042600	-2.35002800	-0.03147000
O	-0.32932600	0.11674000	1.43903400
H	-1.23145300	0.26626200	1.01831200
H	-0.53077200	0.18142400	2.37968300
O	-1.66213000	2.50533400	-0.69888500
H	-2.16689900	1.89405700	-0.11275400
H	-2.33909400	2.96141900	-1.20947100
O	-0.81623300	-0.01941700	-1.71920800
H	-1.49089300	-0.64123700	-1.37953400
H	-1.19395800	0.85381700	-1.52160400

SIP+5W

0 1

C	-3.36716200	1.23256900	-0.45311900
C	-3.76611800	-0.03366500	-0.76292900
C	-1.81097200	-0.16658700	0.24153000
H	-3.84710500	2.19058900	-0.62421000
H	-4.66217000	-0.39683700	-1.25549500
H	-0.88358600	-0.53283400	0.69310200
N	-2.14880500	1.11875000	0.17523000
N	-2.78171500	-0.88853900	-0.31798200
C	-1.29630400	2.21088200	0.65938600
H	-1.82400600	2.70531800	1.48803600
H	-0.39038700	1.74195300	1.05859300
C	-0.92703500	3.18840500	-0.44022600

H	-0.20618200	3.90971500	-0.03440400
H	-0.44390800	2.63854400	-1.25675400
H	-1.79877200	3.73858000	-0.82389300
C	-2.79492800	-2.34838700	-0.42924900
H	-3.66322900	-2.63107400	-1.03392800
H	-1.86358200	-2.67746400	-0.90647000
H	-2.84229400	-2.78391000	0.57467600
C	4.86273500	-0.40507100	-0.80621100
H	5.44133300	0.22273700	-0.11321100
H	5.21899300	-1.43883600	-0.74887200
H	5.01086600	0.00568700	-1.81348300
C	3.39332200	-0.31701100	-0.43344800
O	2.83127300	0.80808800	-0.61989000
O	2.82732500	-1.32174000	0.03231900
O	0.34841100	-2.32011700	-0.73811100
H	0.29600400	-1.38684800	-1.03281700
H	1.29711900	-2.38653300	-0.53467600
O	0.39398600	0.36949500	-1.42761600
H	1.29597100	0.56494600	-1.02626300
H	0.55456100	0.47800300	-2.37225900
O	1.79608300	2.23102900	1.38425200
H	2.24137400	1.81171300	0.60771600
H	2.51224600	2.61773100	1.89809400
O	0.83644900	-0.36476800	1.65002300
H	1.51201000	-0.79100800	1.07247400
H	1.20754000	0.51843000	1.82538100
O	-0.86947300	-2.56819200	1.80762200
H	-0.31363300	-1.90299900	2.24151700
H	-0.40918300	-2.66423800	0.95323400

SIP+6W

0 1

C	3.87713100	0.72750800	-0.16851000
C	4.16128300	-0.57741300	0.10367500
C	1.97191000	-0.36521000	0.00775000

H	4.52905700	1.58120800	-0.32299300
H	5.11044300	-1.08930700	0.22500200
H	0.90224000	-0.56745500	-0.01181900
N	2.50602300	0.83031000	-0.22304900
N	2.95871900	-1.23705800	0.21680500
C	1.72692500	2.06333900	-0.39261300
H	2.04200000	2.52823100	-1.33716300
H	0.67776500	1.76173400	-0.48771000
C	1.90742200	2.99720200	0.78965400
H	1.25573400	3.86618700	0.64463300
H	1.58926400	2.48822200	1.71094100
H	2.95150600	3.32469700	0.90292100
C	2.78947300	-2.67038400	0.44194000
H	3.60221700	-3.01476700	1.09155600
H	1.81702900	-2.82770200	0.92337300
H	2.79610000	-3.18597100	-0.52478400
C	-4.93243300	-1.17461000	0.53617700
H	-5.57051400	-0.54758200	-0.10247700
H	-5.11319500	-2.23290400	0.32233300
H	-5.19202100	-0.93957400	1.57720800
C	-3.48007000	-0.81169000	0.29163300
O	-3.14169900	0.38524000	0.55647700
O	-2.70352400	-1.67540900	-0.15543900
O	-0.30851200	-2.16967700	1.05283500
H	-0.35881300	-1.24987300	1.37322200
H	-1.20966400	-2.29087200	0.69650900
O	-0.70746000	0.62792200	1.50038400
H	-1.59336700	0.52049600	1.03532800
H	-0.96843100	0.82311700	2.40914200
O	-2.61614200	1.86717000	-1.55989700
H	-2.95891100	1.38772200	-0.76280300
H	-3.38500100	2.09860600	-2.09052400
O	-0.71863400	-0.15118400	-1.37007400
H	-1.38270600	-0.74320600	-0.95004300

H	-1.26337300	0.57623800	-1.71803400
O	0.83027800	-2.42488500	-1.68420000
H	0.35750200	-1.63461900	-1.99288500
H	0.38045900	-2.59481000	-0.84230000
O	-0.99822600	3.30922800	0.20798500
H	-0.90035200	2.50697300	0.74140600
H	-1.58667400	3.02275200	-0.50913600
